# pH-Responsive PLGA Nanoparticle for Controlled Payload Delivery of Diclofenac Sodium

**DOI:** 10.3390/jfb7030021

**Published:** 2016-08-02

**Authors:** Shalil Khanal, Udhab Adhikari, Nava P. Rijal, Shanta R. Bhattarai, Jagannathan Sankar, Narayan Bhattarai

**Affiliations:** 1 Department of Energy and Environmental Systems, North Carolina A & T State University, Greensboro, NC 27411, USA; skhanal@aggies.ncat.edu; 2 Engineering Research Center Revolutionized Metallic Biomaterials, North Carolina A & T State University, Greensboro, NC 27411, USA; uadhikar@aggies.ncat.edu (U.A.); nprijal@aggies.ncat.edu (N.P.R.); sankar@ncat.edu (J.S.); 3 Department of Mechanical Engineering, North Carolina A & T State University, Greensboro, NC 27411, USA; 4 Department of Chemical, Biological, and Bioengineering, North Carolina A & T State University, Greensboro, NC 27411, USA; 5 Department of Experimental Radiation Oncology, University of Texas MD Anderson Cancer Center, Houston, TX 77030, USA; sbhattarai@mdanderson.org

**Keywords:** nanoparticle, PLGA, chitosan, diclofenac drug, drug delivery, pH dependent

## Abstract

Poly(lactic-co-glycolic acid) (PLGA) based nanoparticles have gained increasing attention in delivery applications due to their capability for controlled drug release characteristics, biocompatibility, and tunable mechanical, as well as degradation, properties. However, thorough study is always required while evaluating potential toxicity of the particles from dose dumping, inconsistent release and drug-polymer interactions. In this research, we developed PLGA nanoparticles modified by chitosan (CS), a cationic and pH responsive polysaccharide that bears repetitive amine groups in its backbone. We used a model drug, diclofenac sodium (DS), a nonsteroidal anti-inflammatory drug (NSAID), to study the drug loading and release characteristics. PLGA nanoparticles were synthesized by double-emulsion solvent evaporation technique. The nanoparticles were evaluated based on their particle size, surface charge, entrapment efficacy, and effect of pH in drug release profile. About 390–420 nm of average diameters and uniform morphology of the particles were confirmed by scanning electron microscope (SEM) imaging and dynamic light scattering (DLS) measurement. Chitosan coating over PLGA surface was confirmed by FTIR and DLS. Drug entrapment efficacy was up to 52%. Chitosan coated PLGA showed a pH responsive drug release in in vitro. The release was about 45% more at pH 5.5 than at pH 7.4. The results of our study indicated the development of chitosan coating over PLGA nanoparticle for pH dependent controlled release DS drug for therapeutic applications.

## 1. Introduction

Drug delivery devices using biodegradable and biocompatible poly(lactic-co-glycolic acid) (PLGA) based nanoparticles have attracted a great deal of attention, as these systems can provide a sustained and controlled drug release and reduce side effects [1,2,3,4,5]. They can protect drugs from degradation and enhance their stability. Moreover, due to their size, nanoparticles can penetrate specific tissues via (i) the fenestrations present in the endothelium of cancer and inflamed tissue or (ii) via receptors over expressed by target cells [6]. This allows a specific delivery of the loaded drugs, proteins, peptides or nucleic acids to their target tissue [7]. Another major advantage of PLGA over other polymers is that PLGA is approved by the FDA.

Nonsteroidal anti-inflammatory drugs (NSAIDs) are one of the most commonly prescribed drugs in the world [8]. Analgesic and anti-inflammatory effects are exerted by NSAIDs through the inhibition of the cyclooxygenase family of enzymes. A local delivery of anti-inflammatory drugs could be an interesting approach to avoid potential side effects resulting from systemic applications [9]. Diclofenac Sodium (DS) is a NSAID that is indicated in relief of signs and symptoms of ankylosing spondylitis, rheumatoid arthritis, osteoarthritis, migraines and menstrual pain. Diclofenac has been frequently used to treat moderate post-operative or post-traumatic pain. However, the short biological half-life of DS severally reduces its application for the long-term treatment of inflammatory disorders. Long-term continuous infusion or multiple dosing schedules like other NSAIDs, DS also has common side effects like gastrointestinal lesion formation, and renal damage [10]. Several research strategies have been focused to overcome these limitations, when DS drug encapsulated into polymer-based nanoparticles are administered orally [11,12,13]. Biodegradable polymer based nanoparticle; particularly PLGA/drug formulation strategies, can greatly enhance the therapeutic drug concentration in the blood stream for a long period of time and can subsequently reduce the multiple drug dose schedules. However, it is difficult to achieve functionalities, such as active targeting in PLGA-based drug delivery systems, due to deficiency of functional groups on PLGA surface. The negative charge on PLGA surface reduces cell affinity and immobilization of cell-targeting molecules. Therefore, surface modification of PLGA-based drug carriers is necessary for getting best ability of the system [4,14]. Cationic surface modifications of such systems has been accomplished by using cetyltrimethylammonium bromide, polyethyleneimine, poly(2-dimethylamino)ethyl methacrylate, didodecyldimethylammonium bromide, poly (ethylene glycol) and chitosan [4,15,16].

Chitosan (CS) is a cationic and pH responsive polysaccharide produced by deacetylation of chitin. It is a structural element in the exoskeleton of crustaceans and insects [17]. The presence of primary amino groups in chitosan is responsible for several important properties such as in situ gelation, transfection, permeation enhancement, mucoadhesion, and efflux pump inhibitory. Chitosan has been widely used in the field of surface modification due to several desirable properties which includes-mild processing conditions, chemical reactivity, minimal foreign body reaction, and cost effectiveness [18,19,20]. By modifying the surface of PLGA NPs, CS provides the following advantages: (i) a decreased burst effect in encapsulated drug release; (ii) increased stability of macromolecules such as proteins; (iii) enhances the Zeta potential inversion, and promotes cellular adhesion and retention of the delivery system at the target site; and (iv) offers the possibility of conjugating targeting ligands to free amino groups on its surface [21,22,23,24]. Chitosan and its derivative also help in the pH dependent release of drug in different nanoparticle systems [25,26,27,28]. Chitosan modified nanoparticles has been used for drug delivery where it could play dual function for protecting the DS drug against the action of the enzymes and gastric fluids, and reduction of the gastrointestinal irritation caused by DS drugs through pH dependent controlled release system from the PLGA particles.

In this work, we investigated the ability of CS coated PLGA based NPs to entrap and release, a model drug, NSAID diclofenac, in a controlled manner. We synthesized core-shell NPs in which PLGA being core and CS being an external shell on NPs surface. Double-emulsion solvent evaporation technique was used to synthesize CS coated DS loaded nanoparticles (CS-DS-PLGA). Uncoated nanoparticles of PLGA (DS-PLGA) were used as control for comparative study. The physio-chemical and surface properties of the different formulations of CS-DS-PLGA and DS-PLGA were studied. In vitro drug release profiles were evaluated at different pH.

## 2. Results and Discussions

### 2.1. Design and Characterization of PLGA Nanoparticles

**Figure 1:**
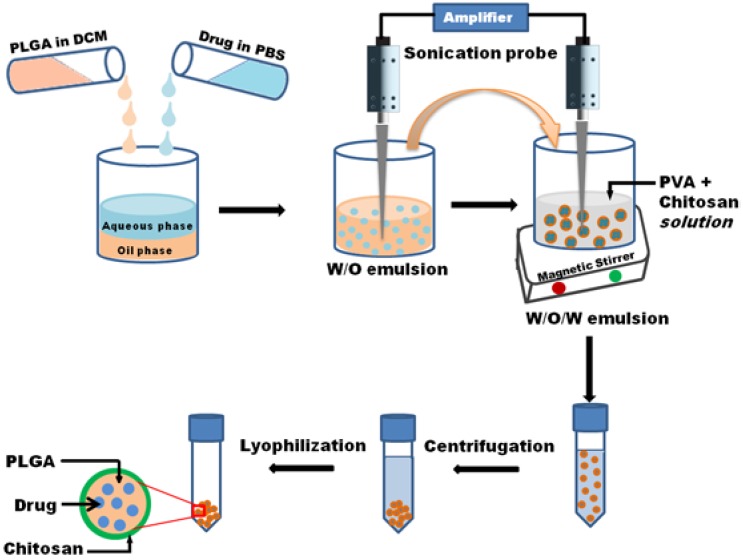
Schematic representation of nanoparticle fabrication.

**Figure 2:**
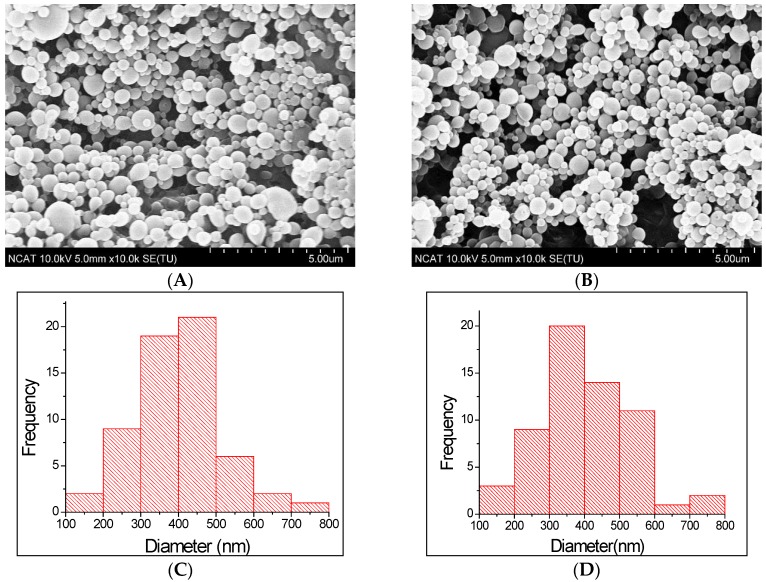
SEM images (**A**,**B**) and size distribution (**C**,**D**) where, (A,C) DS-PLGA; (B,D) CS-DS-PLGA. The microspheres were found to be uniform with some deviations and nominal aggregation.

PLGA particles can be prepared by single- or double-emulsion techniques. These methods provide the ability to customize various particle characteristics including size, encapsulant, and surface properties [29]. In this study, we fabricated the nanoparticles by double emulsion solvent evaporation technique. The schematic representation of the process is shown in Figure 1. Surface morphology and size distribution of prepared nanoparticles were examined through SEM images. Figure 2 shows the surface morphology and size distribution of corresponding nanoparticle. The microspheres were found to be uniform spherical, and have smooth surfaces with some deviations and nominal aggregation. From size distribution study using ImageJ software, the mean diameters of DS-PLGA and CS-DS-PLGA nanoparticles were found as 398.8 and 403.8 nm, respectively. The results obtained from the DLS measurement is shown in Table 1. These results are in agreement in size with the scanning electron microscopic images (Figure 2). Measurements of the particle size showed a slight increase in the diameter (from 415 ± 6 nm to 420 ± 6 nm) by the addition of chitosan. It is found that the chitosan uncoated PLGA nanoparticles have a zeta potential of −14 ± 0.4 mV, which is due to the presence of carboxyl end groups on nanoparticle surfaces. Obviously, after the chitosan treatments, sample shows a positive zeta potential (27 ± 0.6 mV), which indicates the amine groups present in the chitosan successfully coated onto the PLGA nanoparticle surfaces.

Particles of PLGA, smaller than 10 μm and coated with chitosan, have great potential applications in ocular drug delivery. These particles exhibit reduced eye irritation and enhanced corneal penetration with better drug bioavailability compared to larger PLGA particles (>10 μm) [30,31,32]. Additionally, positively charged nanoparticles show prolonged retention times on the corneal surface when compared to negatively charged nanoparticles because of possible electrostatic interactions between the positively charged particles with negatively charged mucin in eyes [30,32].

**Figure 3:**
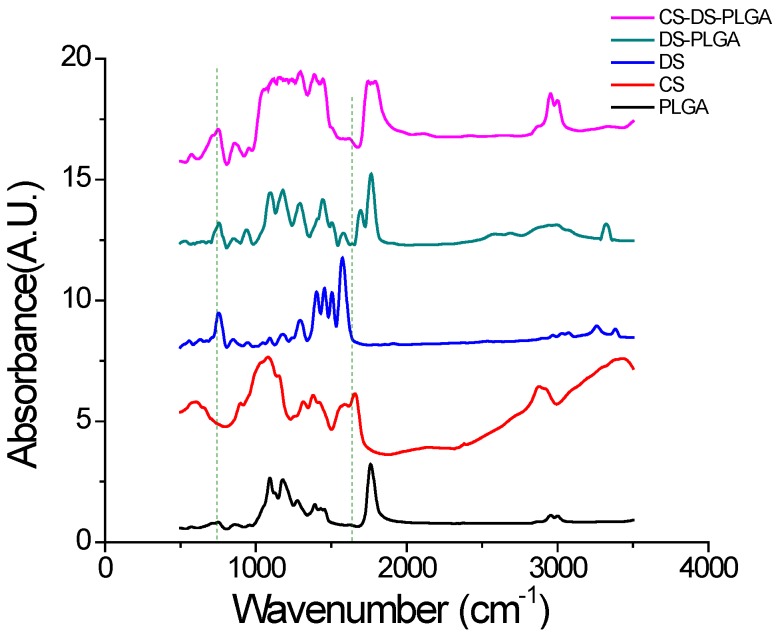
FTIR spectra of PLGA, CS, DS, DS-PLGA, and CS-DS-PLGA. The major peaks for all nanoparticles samples is measured at 1175 cm^−1^ and 1760 cm^−1^ which corresponds to the standard basic measurement of PLGA absorption band. Characteristic absorption peak of chitosan at 1545 cm^−1^ and 1630 cm^−1^ and that of drug at 768 cm^−1^ confirms their presence in respective nanoparticles.

The FTIR spectra confirmed the chitosan adsorption to the surface of PLGA. Figure 3 shows the FTIR spectra of PLGA, CS, DS, DS-PLGA and CS-DS-PLGA. In the PLGA spectrum, the peak of C–O–C stretching is at 1175 cm^−1^, C–H bend is at 1390–1440 cm^−1^, C=O is at 1760 cm^−1^ and C–H stretch is at 3000 cm^−1^. From CS-DS-PLGA spectra, the chitosan adsorption to the surface of PLGA was confirmed by two intense peaks at 1545 and 1630 cm^−1^, corresponding to amine and amide bonds, and broad N–H signal of chitosan at 3200–3700 cm^−1^. Amino peak (N–H stretching), –C=O stretching of the carboxyl ion and C–Cl stretching appeared at 3320, 1573 and 768 cm^−1^, respectively, representing DS in PLGA sample.

**Figure 4:**
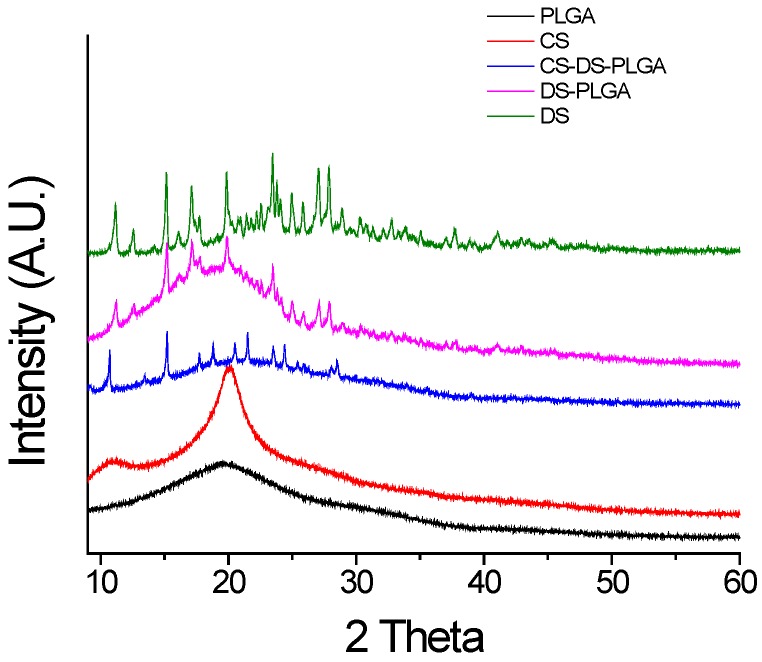
XRD spectra of PLGA, CS, DS, DS-PLGA, and CS-DS-PLGA. The characteristic diffraction peaks of drug at 14.90, 20.74, 25.96, 26.82 and 27.70 at 2θ values are clearly observed in DS-PLGA and CS-DS-PLGA nanoparticles. PLGA nanoparticles and chitosan powder showed no distinct peak which indicates the amorphous nature of these polymers.

The XRD patterns for PLGA, chitosan (CS), diclofenac sodium (DS), DS-PLGA and CS-DS-PLGA are shown in Figure 4. For PLGA and CS, the XRD pattern showed no distinct peak, a fact that indicates, these polymers are amorphous [33,34]. DS powder exhibited crystalline structure with distinct XRD peaks appearing at 14.90, 20.74, 25.96, 26.82 and 27.70 at 2θ values [35]. In the case of the nanoparticles, the principal peaks of the drug existed but could be observed with less intensity, reflecting that the DS existed mostly in an amorphous state and was successfully entrapped into the NPs.

**Figure 5:**
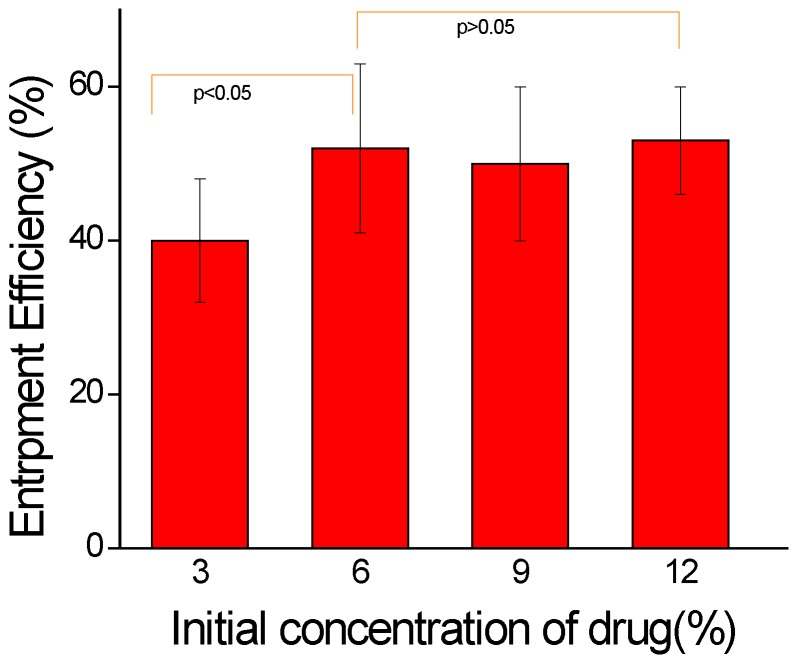
Drug entrapment efficiency of CS-DS-PLGA nanoparticle. Optimum drug entrapment efficiency was found to be 52% with 6% initial drug. After that, there is no significance difference with increasing initial drug concentration (mean ± S.D., *n* = 3).

The relative amount of diclofenac sodium (as compared with the amount of the polymer) was varied between 3% and 12% (wt/wt) to study effect of proportional amount of drug substance on the entrapment efficiency. The result (Figure 5) shows that the entrapment efficiency was increased from 40% to 52% with increased drug amount from 3% to 6% (wt/wt) but, after that there is no significance difference for increased drug amount. Thus, 6% drug was used as optimized condition for further study. The encapsulation efficiencies were low because of the leakage of DS to the external medium, resulting in decreased drug content in the nanoparticles. The result for drug calculation from optimized samples is shown in Table 1. The amount of entrapped DS in PLGA NPs was measured before and after CS coating, which was not significant (*p* > 0.05) for both samples, which suggested that there was no drug leakage during the coating procedure.

### 2.2. Effect of Chitosan Concentration

**Figure 6:**
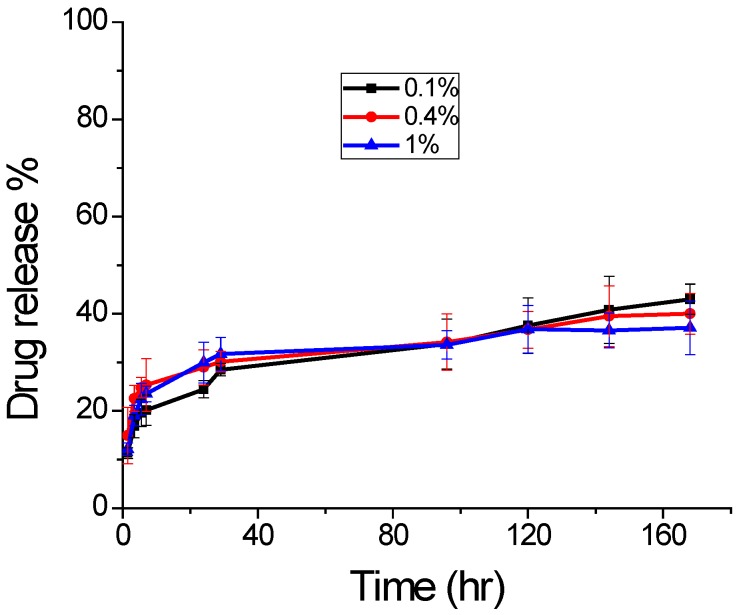
Drug release profile with variable chitosan concentration at pH 7.4 and 37 °C (mean ± S.D., *n* = 3).

The drug release was studied for 7 days from chitosan coated sample, prepared with different concentration to study the effect of concentration change in chitosan. The obtained result is shown in Figure 6. Though all sample showed a burst release of drug initially, the sample with 0.4% chitosan had higher release. However, the overall drug release pattern is similar for all three samples for 7 days with no significant difference. Thus, for further release study, we used the sample from 0.4% chitosan. The overall drug release depends on various factors, such as molecular weight of polymer, amount of drug entrapped, size and overall porosity of the particles. For applications requiring higher release of encapsulated drugs, low molecular weight PLGA can be used, which forms highly porous surface for greater drug release [36].

### 2.3. In Vitro Drug Release

**Figure 7:**
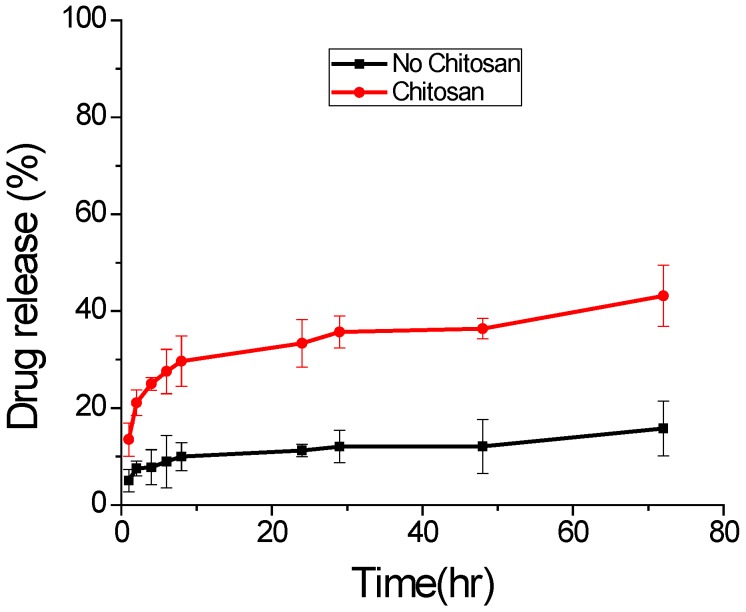
In vitro drug release at pH 7.4 and 37 °C. The chitosan coated sample was prepared with 0.4% chitosan (mean ± S.D., *n* = 3).

In vitro drug release profiles of DS from the treated CS-DS-PLGA nanoparticles and untreated DS-PLGA nanoparticles are shown in Figure 7. Both samples show a burst release in the first 8 h. The modified CS-DS-PLGA nanoparticles exhibited heavier burst release than the untreated nanoparticles because the chitosan and DS have opposite surface charge and some of the drug DS is adsorbed to PLGA nanoparticle surface during the synthesis and solvent evaporation process. Swelling of PLGA polymer, pore diffusion, polymer erosion and degradation contribute to the drug release from both samples [37]. Therefore, after the burst release, both nanoparticles show moderate and sustained release. Chitosan is more hydrophilic than PLGA, which allows PBS solution to more easily penetrate into the nanoparticle matrix and release more drug molecules during the same time. In addition, the presence of positively charged chitosan on the surface of nanoparticles attracts the negatively charged DS drug towards it, thereby facilitating a faster release. Both of these factors contribute to a higher cumulative drug release from PLGA-CS nanoparticles [4].

### 2.4. Effect of pH on Drug Release

**Figure 8:**
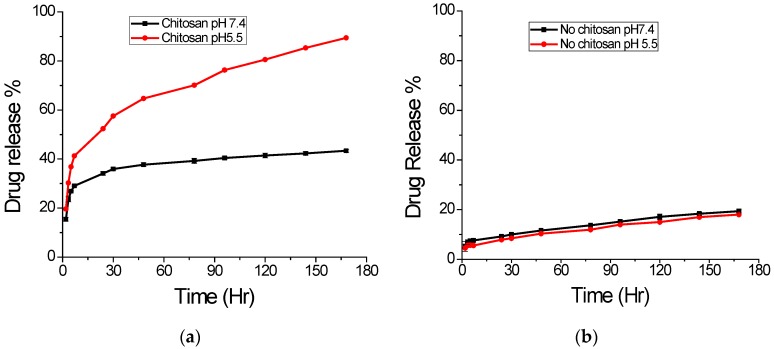
Effect of different pH on drug release at 37 °C. (**a**) Chitosan coated; (**b**) Chitosan uncoated (mean ± S.D., *n* = 3).

The pH dependent in vitro drug release from nanoparticles was carried out in two different buffered solutions with pH 7.4 and 5.5 at 37 °C. The in vitro release profile of DS from different NPs is presented in Figure 8. It shows biphasic character with a brisk initial burst release followed by a sustained release. The graph in Figure 8a shows the release profiles obtained for chitosan uncoated PLGA NPs. The release pattern is similar, but only about 20% release in 7 days for both pH values, which suggests pH independent nature of this sample. As can be seen, both release profiles exhibited rapid burst effect during the first 8 h, indicating no chemical interactions between DS and the polymeric chains [21]. However, in case of chitosan-coated PLGA nanoparticles, the release profile is highly different, as shown in Figure 8b. For pH 5.5, there is nearly 90% release of drug, but the same sample the release is only about 43% at pH 7.4 within 7 days. This result indicates the pH dependent nature of chitosan coated sample as expected due to higher solubility of chitosan in acidic pH. Chitosan has a primary amine group in its glucosamine monomeric unit with low pKa value (6.3), which is protonated and positively charged at low pH, making chitosan soluble in aqueous solution. At higher pH (alkaline region), this amine group becomes deprotonated and uncharged, hence making insoluble biopolymeric hydrogel networks.

## 3. Materials and Methods

### 3.1. Materials

Poly(lactic-co-glycolic acid) (PLGA, lactic acid/glycolic acid = 50:50, with ester end groups, inherent viscosity (0.26–0.54 dL/g)) was purchased from Durect Corporation (Birmingham, AL, USA). Chitosan (medium Mw), poly (vinyl alcohol) (PVA, ~99% hydrolyzed), diclofenac sodium drug was purchased from Sigma Aldrich (St. Louis, MO, USA). Dichloromethane (DCM, ~99%), glacial acetic acid and sodium hydroxide (NaOH) were purchased from Acros Organics (Morris Plains, NJ, USA). Phosphate buffered saline (PBS, 1× solution) and acetate buffer (pH 5.5) were purchased from Thermo Fisher Scientific (Fair Lawn, NJ, USA).

### 3.2. Nanoparticle Preparation

PLGA nanoparticles (NP) were prepared by a single/double emulsion solvent evaporation technique with some modifications [38]. Briefly, 200 mg of PLGA was dissolved in 2 mL of DCM and added drop wise to 5 mL of 0.3% PVA solution under constant vortex. The solution was emulsified in ice bath for 2 min by using Ultra-Sonicator (Thermo Fisher Scientific, Fair Lawn, NJ, USA) at 40% amplitude with 10 s on/off pulse mode. Then, the emulsified solution was transferred into 45 mL of 0.3% PVA solution and the mixture was stirred for 3 h to allow the solvent evaporation and particle hardening. The particles were collected by Sorvall Stratos Centrifuge (Thermo Fisher Scientific, Fair Lawn, NJ, USA) at 10016× *g* for 30 min at 4 °C and washed three times with DI water. The purified PLGA NPs were lyophilized for 2 days by using Freezone Freeze Dryer (Labconco, Kansas City, MO, USA) and stored at −20 °C.

For drug encapsulation, the diclofenac drug solution was mixed with polymer solution and the mixture was sonicated for 30 s before mixing with PVA emulsifier as above. The chitosan coated samples were prepared by mixing the chitosan solution (in 1% Acetic acid) with PVA solution before adding the emulsified polymer solution for stirring.

### 3.3. Morphology Study

The surface morphology of the nanoparticles was observed by scanning electron microscope (SEM) (Hitachi SU8000, Hitachi High-Technologies Corporation, Tokyo, Japan). Specimens were attached to the sample holder by using double sided carbon tape. Prior to imaging with the SEM, samples were sputter coated with gold under vacuum by using Polaron SEM Coating System (Quorum Technologies, East Sussex, UK) for 2 min. The images were acquired at an accelerating voltage of 10 kV and current of 5 μA.

The size distribution of the nanoparticles was analyzed through SEM images with the use of ImageJ software (NIH, Bethesda, MD, USA). Physical length was converted to pixels with the help of scale bar. Seventy-five nanoparticles of each group (*n* = 3) were measured in pixels. Average size and standard deviation were calculated based on converted ImageJ data.

### 3.4. Size Distribution and Zeta Potential

The NPs size and zeta potential were measured by using dynamic light scattering technique (DLS) on Zetasizer Nano ZS (Malvern Instruments, Malvern, UK). The freshly prepared and purified suspension of NPs was diluted in deionized (DI) water and the mixture was vortexed for 2 min. The analysis was carried out at a scattering angle of 90° and a temperature of 298 K. The Z-average diameter and zeta potential were calculated using Malvern software. Zeta potential, based on the electrophoretic mobility was measured to estimate the surface charge. For statistical analyses, three different readings were taken.

### 3.5. Fourier Transform Infrared (FTIR) Analysis

FTIR was used to analyze the chemical interactions among PLGA, chitosan and DS. Absorbance spectra were recorded at 200 scans using IR Prestige21 FTIR Spectrometer (Shimadzu, Kyoto, Japan) from wavenumbers of 400 to 4000 cm^−1^ with a resolution of 4 cm^−1^. A pellet was made for FTIR measurement by dispersing the nanoparticles in KBr matrix and compressing at high pressure.

### 3.6. X-Ray Diffraction (XRD) Study

Crystallography and phases of different nanoparticles were examined by using a Bruker AXS D8 Discover X-ray diffractometer (Bruker Daltonics Inc., Billerica, MA, USA) with Cu-Kα radiation. The X-ray diffraction experiments were performed using a locked-coupled scan with a scanning range (diffraction angle, 2θ) set between 10° and 60°. The instrument was operated in the continuous mode, in increments of 0.0146°, 2θ. All experiments were performed at room temperature.

### 3.7. Entrapment Efficiency and Drug Content

The entrapment efficiency and drug content of DS loaded nanoparticles was analyzed according to previous methods [36,39]. Briefly, 50 mg of NPs containing drug were dissolved by adding 5 mL of DCM and the polymer was precipitated after the addition of methanol. During the synthesis of nanoparticles, the amount of drug in the supernatant after each step of centrifugation was quantified. A calibration curve was developed using the known standard concentrations of DS dissolved in DI water. The spectrophotometric quantification was achieved by taking the absorbance at 277 nm. The encapsulation efficiency was calculated using the formula:

$$EE\text{ \%}=\frac{\text{Total amount of drug used }–\text{ amount of drug in the supernatant}}{\text{Total amount of drug used}}\;\times \;100$$

Then, percentage of drug content was calculated using the formula:

$$\text{Experimental drug loading }=\frac{L}{\text{Lo}}\;\times \;100$$

where L is the actual drug content and Lo is the weighed quantity of beads.

### 3.8. Study of Effect of Chitosan Concentration on Drug Release

Three different samples were prepared by using different concentration (0.1%, 0.4% and 1%) of chitosan by same technique as described above. In order to study, 60 mg of each sample was added to 3 mL of 1X PBS solution and incubated in a Shaking Incubator (Dubnoff Shakebath-2876, Thermo Fisher Scientific, Fair Lawn, NJ, USA) at 37 °C and 50 rpm. At different time intervals, aliquots of 2 mL were collected from each sample solution and added same volume of fresh PBS to keep the final volume constant throughout the study. Then, released drug concentration was calculated by using a UV-Spectrophotometer (GENESYS 10S, Thermo Fisher Scientific, Fair Lawn, NJ, USA) at a wavelength of 277 nm. Since there is no significant difference in drug release from sample with different chitosan concentration a further test was performed with a sample prepared by using 0.4% chitosan.

### 3.9. In Vitro Drug Release Study

In order to investigate the release of Diclofenac from DS-PLGA and CS-DS-PLGA NPs, a fixed amount (50 mg) of each formulation was incubated in 6 ml of 1× phosphate buffer solution (PBS, pH 7.4) at 37 °C and 50 rpm. At fixed time intervals, 5 mL of the supernatant was withdrawn and replaced with fresh buffer. The absorbance of each solution was measured at 277 nm and compared with a calibration curve to determine the amount of drug released. All experiments were performed in triplicate.

### 3.10. Determination of Effect of pH on Drug Release

Two different buffer solutions: 1X PBS (pH 7.4) and Acetate Buffer (pH 5.5) were used to study the effect of pH on drug release. Additionally, the release study was performed with a similar method as that described in Section 3.8.

### 3.11. Statistical Analysis

All tabulated results were expressed as mean ± S.D. Data were analyzed for significance with OriginPro software (Origin Lab, Northampton, MA, USA) using one-way analysis of variance (ANOVA). Post hoc Tukey’s test was performed with ANOVA for multiple comparisons. The α-value was set to 0.05 and *p*-values less than 0.05 were considered statistically significant.

## 4. Conclusions

Chitosan coated NPs were successfully synthesized by physical adsorption of its polymeric chains on the surface of drug-loaded PLGA polymeric particles. A physical approach for studying DS release from polymeric NPs (average diameter 390–420 nm), has been proposed. CS coating influenced higher release of DS and pH dependent behavior for PLGA nanoparticles. This release profile could be of potential interest for drug delivery in in vivo human therapies, by producing a constant and uniform concentration of drug inside cells with their long-term biochemical effects. The results of our study indicate the development of chitosan coating over PLGA nanoparticle for pH dependent controlled release DS drug for therapeutic applications.

## Figures and Tables

**Table 1:** Size distribution and Zeta potential of NPs.

Sample	Size (nm) (Mean ± S.D)	Zeta Potential (mV) (Mean ± S.D)	Drug Contain (%)
DS-PLGA	415 ± 6	−14 ± 0.4	3.92
CS-DS-PLGA	420 ± 6	27 ± 0.6	3.13
